# Vaginal Progesterone Effects on Ultrasound Indices, Fetal Outcomes, and Preeclampsia in High-risk Pregnancy: Vaginal Progesterone in High-risk Pregnancy: Ultrasound Indices and Outcomes

**DOI:** 10.31661/gmj.v14i.3745

**Published:** 2025-07-08

**Authors:** Satinik Darzi, Sahereh Arabian, Parvin Motamedi Kia, Sajjad Rahimi Pordanjani, Elham Saffarieh

**Affiliations:** ^1^ Abnormal Uterine Bleeding Research Center, Semnan University of Medical Sciences, Semnan, Iran; ^2^ Semnan University of Medical Sciences, Semnan, Iran; ^3^ Department of Public Health, Behbahan Faculty of Medical Sciences, Behbahan, Iran

**Keywords:** Preeclampsia, Progesterone, Aspirin, Uterine Artery

## Abstract

**Background:**

According to the high prevalence and importance of preeclampsia and its relationship with uterine artery resistance, the purpose of this study was to determine the effect of vaginal progesterone administration on ultrasound indices, fetal outcomes, and high-risk women pregnancy in terms of the incidence of preeclampsia.

**Materials and Methods:**

In this randomized, double-blind clinical trial, the number of 60 pregnant women between 11 to 14 weeks with risk factors for preeclampsia were examined according to the inclusion criteria and based on random assignation method in two groups of 30 patients (intervention group: 80 mg aspirin tablets + 400 mg vaginal progesterone suppositories and control group: 80 mg aspirin tablets) based on maternal and fetal outcomes and uterine artery color Doppler ultrasound (pulsatility index (PI) and vascular resistance index (RI).

**Results:**

The prevalence of preterm birth in the intervention group was lower significantly difference (P≤0.05). In the intervention group, uterine artery PI after the study had a greater decrease than before the study on the right side (0.33±0.42 vs. 0.05±0.28, P≤0.05) and on the left side (0.38±0.49 vs. 0.09±0.2, P≤0.05), compared to the control group. In the intervention group, uterine artery RI after the study had a greater decrease than before the study on the right side (0.20±0.31 vs. 0.02±0.10, P≤0.05) and on the left side (0.22±0.3 vs. 0.03 ± 0.1, P≤0.05), compared to the control group.

**Conclusion:**

Progesterone suppositories in addition to aspirin, can reduce the prevalence of preterm birth and uterine artery PI and RI values.

## Introduction

**Figure-1:**
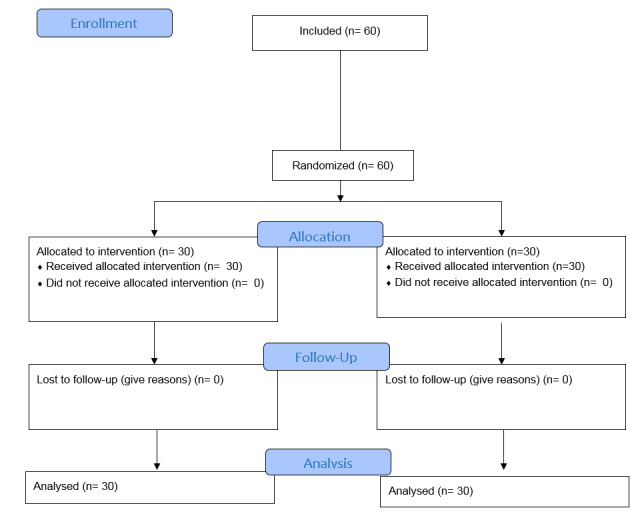
Figure 1. CONSORT Flow Diagram of the study

Hypertensive disorders that occur during pregnancy course, are common disorders and along with hemorrhage and infection, are considered three deadly factors; these factors are responsible for a major part of maternal mortality and complications, which one of the most important of them, is preeclampsia disorders [1][2]. In general, gestational hypertension and preeclampsia are affected 18 million pregnant women worldwide annually [3][1]. Preeclampsia disorders are associated with high maternal, fetal, and neonatal complications; among the maternal complications of preeclampsia, can be implied to renal necrosis, pulmonary edema, liver necrosis, hemolysis, increased liver enzymes, thrombocytopenia, and cerebrovascular accidents [4][5].

Regarding neonatal complications of preeclampsia disorder, can be implied to stillbirth, neonatal death, intraventricular hemorrhage, hypoxic ischemic encephalopathy, low Apgar score at 5 minutes, neonatal seizures, respiratory distress syndrome, pneumothorax, and necrotizing enterocolitis [5][6][7]. The definitive treatment for preeclampsia and other blood pressure disorders in pregnancy, is delivery and placental abruption; but on the one hand, premature birth entails risks that are not completely eliminated even with the use of corticosteroids within 24 to 48 hours before delivery, and on the other hand, pregnant women with early preeclampsia are exposed to severe consequences and even death [6]. Currently, there is no definitive and effective treatment for preeclampsia and prevention of it, has great importance. In this regard, the administration of low-dose aspirin has been proposed in the prevention of preeclampsia. Aspirin, by inhibiting the synthesis of thromboxane A2, leads to maintain resistance to angiotensin II and, consequently, reduces uterine artery resistance [8].

Although the initial successes in reducing the incidence of preeclampsia has been reported with the administration of low-dose aspirin in suppressing thromboxane A2 and the superiority of prostacyclin, but several clinical trials have reported the ineffectiveness of aspirin in preventing the incidence of preeclampsia, especially in cases with high uterine artery resistance [9][10].

The studies have shown that the administration of progesterone can help reduce the incidence of preeclampsia in these patients [11][12]. Progesterone may participate in the regulation of vascular tone by inhibiting platelet aggregation, but the actual mechanism that causes the incidence of vasodilatory effects, has remained unknown. The effects of progesterone are mediated through the cyclic adenosine monophosphate (cAMP) mechanism and may be physiologically important in maintaining low vascular resistance and providing adequate blood flow in the placental circulation, and through this mechanism may be effective in preventing preeclampsia [13][14].

In addition, reducing the production of prostaglandins and also preventing the activity of contractile proteins are considered as the possible causes of the improvement of uterine blood flow in the second half of pregnancy by progesterone [15]. Therefore, the purpose of this study was to investigate the effect of vaginal progesterone administration on ultrasound indices, fetal outcomes, and high risk female's pregnancy in terms of the incidence of preeclampsia.

## Materials and Methods

**Table:** Table1. The Distribution of the Demographic and Background Information in Pregnant Women with Preeclampsia Risk Factors based on the Studied Group

**Demographic and background information**		**Intervention**		**Control**		**P-value**
		**Number**	**Percentage**	**Number**	**Percentage**	
**Age group (years)**	Young (<29)	9	0.3	7	323	0.559
	Middle-aged (30 - 59)	21	0.7	23	76.7	
	Thin (<18.5)	0	0	2	6.7	
**Body mass index (kg/ m^2^) **	Normal (18.5-24.9)	4	13.3	3	0.1	0.507
	Overweight (25 -29.9)	11	36.7	12	0.4	
	Obese	15	0.5	13	43.3	
**History of abortion**	Yes	12	0.4	13	43.3	0.255
	No	18	0.6	17	56.7	
**Type of birth**	Normal	11	36.7	13	43.3	0.598
	Cesarean section	19	63.3	17	56.7	

**Table:** Table2. The Distribution of the Prevalence of Preeclampsia Risk Factors in Pregnant Women with Preeclampsia Risk Factors based on the Studied Group

**Risk factors for preeclampsia**		**Intervention**		**Control**		**P-value**
		**Number**	**Percentage**	**Number**	**Percentage**	
**History of preeclampsia**	Yes	7	23.3	4	13.3	0.317 *
	No	23	76.7	26	86.7	
**Previous history of preeclampsia in mother or sister**	Yes	9	0.3	7	23.3	0.559 *
	No	21	0.7	23	76.7	
**Multiple pregnancy**	Yes	1	3.3	1	3.3	0.754 **
	No	29	96.7	29	96.7	
**Chronic hypertension**	Yes	10	33.3	12	0.4	0.592 *
	No	20	66.7	18	0.6	
**Pre-gestational diabetes**	Yes	4	13.3	8	26.7	0.197*
	No	26	86.7	22	73.3	
**Kidney disease**	Yes	1	3.3	4	13.3	0.177*
	No	29	26.7	26	86.7	
**Autoimmune disease**	Yes	2	6.7	1	3.3	0.5**
	No	28	93.3	29	96.7	
**Nulliparity**	Yes	7	23.3	8	26.7	0.766*
	No	23	76.7	22	73.3	
**Interval between pregnancies ≥ 10 years**	Yes	12	0.4	8	26.7	0.273*
	No	18	0.6	22	73.3	
**Poor socioeconomic status**	Yes	3	0.1	3	0.1	0.655**
	No	27	0.9	27	0.9	
**Poor pregnancy outcome**	Yes	11	36.7	7	23.3	0.26*
	No	19	63.3	23	76.7	

^*^Chi-square test ^**^Fisher's exact test

### Type of Research and Studied Population

This study is based on a double-blind clinical trial. The studied population includes all pregnant women in the 11th to 14th weeks of pregnancy with one of the high risk factors for preeclampsia who referred to Amir al-Momenin Hospital in Semnan (Iran) between 2022 and 2023.

### Sampling Method and Sample Size

In this study, sampling was carried out by using of convenient or available method. The statistical sample size was estimated with the sample size equal to 20 using the formula for calculating the minimum sample size to compare averages in two independent populations, considering a confidence level of 95% and a power value of 80%. According to the probability loss of 15% of samples, finally, 30 people in each group (Totally 60 people) were examined (Figure-1).

### Inclusion and Exclusion Criteria

The inclusion criteria into the study are as follow: age 18 years and above are one of the risk factors for preeclampsia which includes a previous history of preeclampsia, multiple pregnancy, chronic hypertension, pre-pregnancy diabetes, kidney disease, and autoimmune diseases, or two or more minor criteria including: age above 35, nulliparity, body mass index over 30 kg/m2, interval between pregnancies more than 10 years, history of preeclampsia in the mother or sister, poor socioeconomic status, and poor pregnancy outcome such as stillbirth, LBW, and SGA. Also, the exclusion criteria from the study are as follow: lack of follow-up and repeat visits, heart, liver, thyroid, peptic ulcer diseases, history of asthma, sensitivity to aspirin or progesterone compounds and their use in recent pregnancy, threaten to abortion such as vaginal bleeding and eclampsia or preeclampsia during the time of the study.

### Data Collection Tool

In this study, the data were collected using a researcher-made checklist, which consisted of two sections of demographic information including patient age, history of previous abortion, body mass index, and clinical information including risk factors for preeclampsia, pregnancy outcomes including preeclampsia, intrauterine growth restriction (IUGR), preterm birth (before 37 weeks of pregnancy), fetal outcomes including birth weight, Apgar score, and type of delivery, and uterine artery color Doppler ultrasound. The validity and reliability coefficient of the aforementioned questionnaire was calculated based on Cronbach's alpha, 0.94, which indicates the appropriate validity of this questionnaire.

### Work Method

In this double-blind study, after the approval of the plan at Semnan University of Medical Sciences and Health Services and the approval of the Ethics Committee in Medical Research to the ethics code with No (IR.SEMUMS.REC.1402.125) , this trial registered at IRCT (No 73118) , 60 pregnant women who had all the inclusion criteria were included in the study after obtaining a written consent referred to women's clinics of Amir al-Momenin Hospital (AS) in Semnan during the period of 2022 and 2023 in the 11th to 14th weeks of pregnancy. Then, based on the random assignment method using the randomized block method, 30 pregnant women were assigned to Group A or the intervention group, which were treated with 80 mg of oral enteric-coated aspirin tablets (Cardiosprin 80 mg, manufactured by Samisaz Company, Iran) daily along with 400 mg of vaginal progesterone suppositories (Cyclogest 400 mg, manufactured by Actoveerco Company, Iran) daily from the 12th week of pregnancy for 6 weeks. Group B included the control group, which was treated with 80 mg of oral enteric-coated aspirin tablets (Cardiosprin 80 mg, manufactured by Samisaz Company, Iran) daily from the 12th week of pregnancy for 6 weeks. Blinding method was performed in a double-blind method, so that both the patient and the specialist physician of the patients and the radiologist were unaware of the type of patients' grouping and the medication consumed by each group. In this study, uterine artery RI and PI, which were related to aspirin and progesterone use, were examined immediately before and 6 weeks after the onset of the intervention, then the pregnancy outcomes and fetal outcomes related to uterine artery RI and PI were assessed until the termination of pregnancy. The results were recorded in a researcher-made checklist and statistically analyzed.

### Data Analysis Method

The obtained data were statistically analyzed using IBM SPSS Statistics for Windows, version 26 (IBM Corp., Armonk, N.Y., USA).

The normal distribution of continuous variables was evaluated using the Schroepfer-Wilk test and the data were reported as mean ± standard deviation or median ± 95% central values. The relationship between variables was examined using the t-test for parametric data or Mann-Whitney for nonparametric data. The chi-square test was also used to examine qualitative characteristics. In addition, post-hoc power analysis was conducted for key outcome variables with statistically significant differences between groups.

### Ethical Considerations

All possible complications were explained to the patients in simple and understandable language and the patients were able to withdraw from the plan at any time and for any reason. Participation in the study did not mean deprivation of treatment and did not involve any additional cost for the patients. All information will remain completely confidential by the researcher and the researcher is committed to keeping their information.

## Results

**Figure-2:**
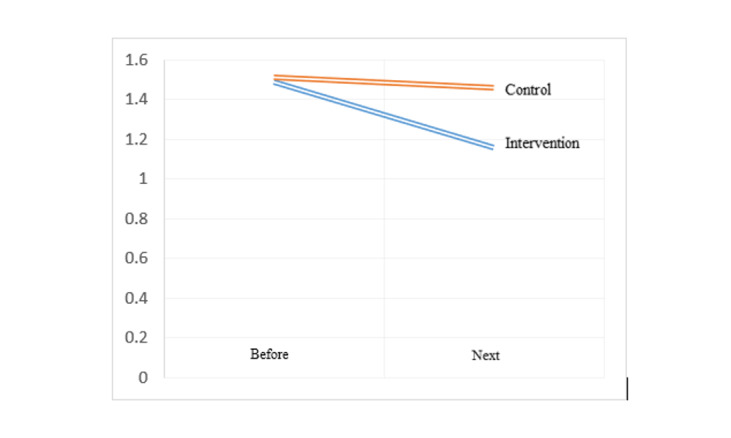
Figure 2. The distribution of right uterine artery RI results in pregnant women with preeclampsia risk factors based on the studied group

**Figure-3:**
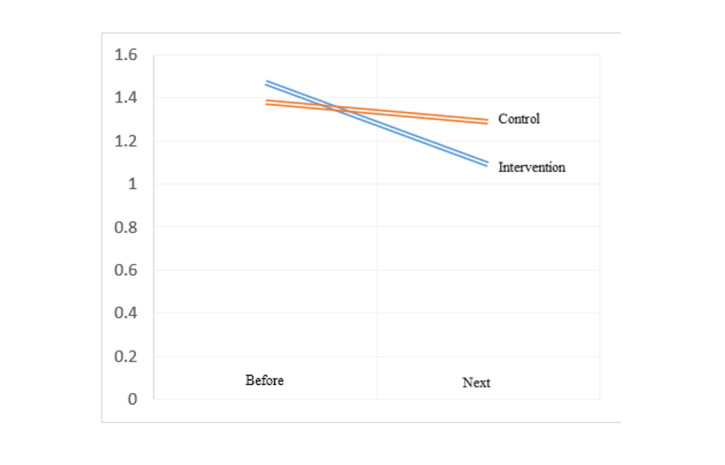
Figure 3. The distribution of left uterine artery RI results in pregnant women with preeclampsia risk factors based on the studied group

**Table:** Table3. The Distribution of Uterine Artery PI Results in Pregnant Women with Preeclampsia Risk Factors based on the Studied Group

**Uterine artery PI**		**Intervention**		**Control**		P-value ^*^
		**Mean**	**Standard deviation**	**Mean**	**Standard deviation**	
	Before study	1.49	0.5	0.51	0.5	0.918
**Right side**	After study	1.16	0.35	0.46	0.46	0.005
	Before and after difference	0.33	0.42	0.05	0.28	0.001
	P-value** for within group comparison		0.326		>0.001	
	Before study	0.47	0.53	0.38	0.37	0.734
**Left side**	After study	1.09	0.29	0.29	0.39	0.043
	Before and after difference	0.38	0.49	0.09	0.2	0.001

^*^Mann-Whitney test ^**^Paired t-test

**Table:** Table4. The Distribution of Left Uterine Artery RI Results in Pregnant Women with Preeclampsia Risk Factors based on the Studied Group

**Uterine artery RI**		**Intervention**		**Control**		P-value ^*^
		**Mean**	**Standard deviation**	**Mean**	**Standard deviation**	
	Before study	0.65	0.22	0.67	0.13	0.97
**Right side**	After study	0.28	0.43	0.65	0.14	0.003
	Before and after difference	0.37	0.31	0.02	0.1	0.046
	P-value** for within group comparison		0.183		>0.001	
	Before study	0.57	0.2	0.64	0.11	0.225
**Left side**	After study	0.26	0.37	0.61	0.12	0.001
	Before and after difference	0.31	0.03	0.03	0.1	0.049

^*^Mann-Whitney test ^**^Paired t-test

The investigation and analysis of demographic information in the women of the intervention and control groups using the chi-square test showed that the mean age in the intervention group was equal to 33.07 ± 6.33 years and in the control group was equal to 33.67 ± 5.94 years which there was observed no significant difference in terms of age in both two groups (P=0.559). The mean and standard deviation of BMI in the intervention group was equal to 29.40 ± 4.23 kg/ m2 and in the control group was equal to 28.91 ± 5.10 kg/ m2 (P=0.507). About 21 patients (70%) of the intervention group and 23 patients (76.7%) of the control group were placed in the middle-aged age group. Among the women participating in the intervention group, 12 women (40%) and 13 women (43.3%) of the control group had a history of abortion (P=0.255). The analysis of demographic information showed that there was no statistically significant difference between the intervention and control groups in terms of BMI and history of abortion (P>0.05, Table-1). Among the studied women, 7 women (23.3%) in the intervention group and 4 women (13.3%) in the control group had a previous history of preeclampsia, but there was observed no statistically significant difference between the two groups in terms of previous history of preeclampsia (P=0.317, Table-2). The most common risk factor for preeclampsia, was an interval between the pregnancies of 10 years or above (0.40%) in the intervention group and chronic hypertension (0.40%) in the control group. However, there was observed no significant difference between the two studied groups in any of the risk factors for preeclampsia (P>0.05). A post-hoc power analysis was performed for the comparison of preeclampsia history between the control and intervention groups (13.3% vs. 23.3%). Using a two-sided test with α=0.05 and 30 participants per group, the calculated statistical power was approximately 20%. Among the studied maternal outcomes, only the prevalence of preterm birth in the intervention group was lower significant difference than the control group (23.3 vs. 0.50%, P≤0.05). Given the major role of preterm birth in neonatal morbidity and mortality, this finding is considered clinically significant, suggesting a potential protective effect of vaginal progesterone. However, eclampsia was not reported in any of the studied patients. Also, in terms of newborn weight (2605.9±628.8 vs. 2691.1±729.6 g and P=0.421), one-minute Apgar (8.53±0.63 vs. 8.7±0.65 and P=0.151) and fifth-minute Apgar (9.67±0.61 vs. 9.80 ±0.48 and P=0.343), there was observed no significant difference in the intervention group compared to the control group (P≥0.05). Furthermore, the magnitude of these differences was minimal and likely not clinically meaningful. In the examination of the right and left uterine arteries PI, in the intervention group compared to the control group, the uterine artery PI values after the study were significantly lower difference on the right side (1.16 ± 0.35 vs. 1.46±0.46, P≤0.05) and on the left side (1.09±1.29 vs. 1.29±0.39, P≤0.05). Also, in the intervention group, the uterine artery PI values after the study compared to before the study on the right side (0.33 ± 0.42 vs. 0.05±0.28, P≤0.05) and on the left side (0.38±0.49 vs. 0.09±0.2, P≤ 0.05) had a greater decrease compared to the control group (Table-3, Figure-2 and Figure-3). A post-hoc power analysis was conducted for the comparison of the right uterine artery PI after the study between the intervention and control groups (mean difference: 0.70, pooled SD:0.41). This yielded a very large effect size (Cohen’s d ≈ 1.71), corresponding to a statistical power of approximately 99% at α=0.05. This indicates that the study was well powered to detect this difference. The observed reductions in uterine artery PI on both sides in the intervention group were not only statistically significant but also clinically meaningful, with large effect sizes (Cohen’s d ≈ 1.71) and high statistical power (~99%). These changes suggest improved uteroplacental perfusion, which could contribute to better maternal-fetal outcomes.

**Figure-4:**
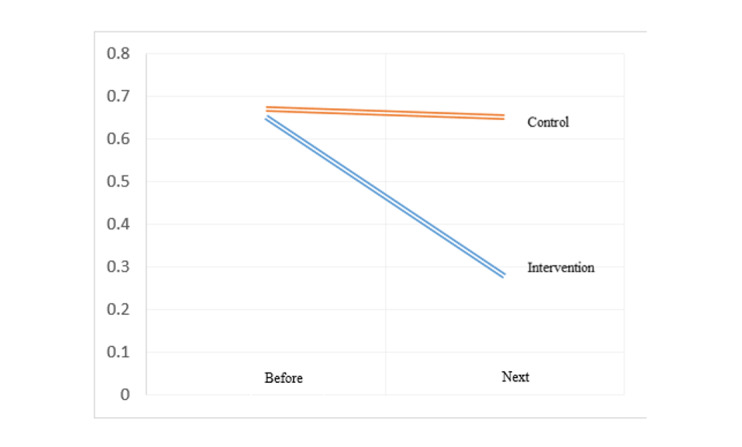
Figure 4. The distribution of right uterine artery RI results in pregnant women with preeclampsia risk factors based on the studied group

**Figure-5:**
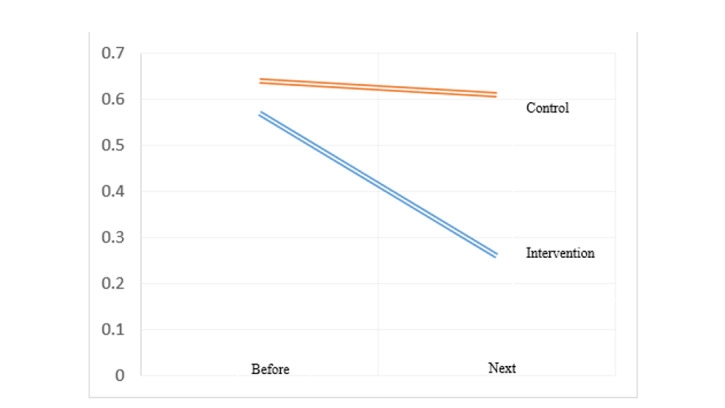
Figure 5. The distribution of left uterine artery RI results in pregnant women with preeclampsia risk factors based on the studied group

On the other hand, in the examination of the right and left uterine arteries PI, in the intervention group compared to the control group, the uterine artery RI values after the study were significantly lower difference on the right side (0.28 ± 0.43 vs. 0.65 ± 0.14 and P ≤ 0.05) and on the left side (0.26 ± 0.37 vs. 0.61 ± 0.12 and P ≤ 0.05). Also, in the intervention group, the uterine artery RI values after the study compared to before the study on the right side (0.37 ± 0.31 vs. -0.02 ± 0.10, P ≤ 0.05) and on the left side (0.31 ± 0.30 vs. -0.03 ± 0.10, P ≤ 0.05) had a greater decrease compared to the control group (Table-4, Figure-4 and Figure-5). For the comparison of left uterine artery resistance index (RI) after the study, the intervention group showed a significant reduction compared to the control group (mean difference: 0.35, pooled SD: 0.275). A post-hoc power analysis indicated a large effect size (Cohen’s d ≈ 1.27), yielding a statistical power of approximately 96% at α=0.05. This suggests the study was adequately powered to detect this difference.

## Discussion

The vaginal progesterone administration and the examination of maternal outcomes in high-risk pregnant women in terms of preeclampsia showed that among maternal outcomes, only the prevalence of preterm birth in the intervention group was significantly lower difference than the control group. According to a study which was conducted by Maged et al. (2020) in Egypt, it was showed that administering 200 mg of vaginal progesterone twice a day can significantly reduce the prevalence of preterm birth in the studied pregnant women [16]. However, according to a study which was conducted by Zhang et al. (2021) in Turkey, it was showed that there was no relationship between first-trimester serum progesterone levels and the prevalence of preterm birth [17].

The reason for this difference can be related to this issue that according to a study which was conducted by Zhang et al. (2021) in Turkey, only the serum progesterone levels of pregnant women in the first trimester were examined descriptively. Pakniat et al. (2021) in Qazvin (Iran) were paid to compare the effect of dydrogesterone 10 mg twice daily and vaginal progesterone 400 mg daily on the final outcome of pregnancy in cases of threatened abortion. In this study, Pakniat stated that the administration of different forms of progesterone had no effect on fetal and neonatal outcomes, including newborn weight [18]. Also, Maged et al. (2020) in Egypt showed that the vaginal progesterone administration had no significant effect on the prevalence of fetal and neonatal outcomes [16].

However, according to a study which was conducted by Zhang et al. (2021), showed that there was no significant relationship between serum progesterone levels and perinatal complications [17]. In our study, there was observed no significant difference between fetal and neonatal outcomes in the intervention and control groups which these results were consistent with the results of Pakniat and Maged studies. However, these results were different from the results of Movahed et al. (2019). The results of Movahed et al. showed that administering 400 mg of vaginal progesterone suppositories for 14 days significantly increased the newborn weight compared to the control group [19].

The reason for this difference could be related to differences in demographic characteristics as well as differences in other measurement tools and methods used in different studies. In the examination of PI values in the intervention group compared to the control group, PI values were significantly lower difference after the study. Also, in the intervention group, PI values had a greater decrease after the study than before the study compared to the control group. According to a study which was conducted by Xie et al. (2023) in Egypt, it was showed that the role of vaginal progesterone on uterine artery Doppler changes in pregnant women at risk of preterm birth was investigated which the results of this study showed that there was a statistically significant difference in uterine artery PI before and after treatment with vaginal progesterone; so that this index decreased significantly after treatment compared to before treatment; since that Xie et al. stated that uterine artery PI decreased significantly after treatment with progesterone compared to before treatment in different periods of pregnancy (weeks) which was consistent with the results of our study [20]. However, in contrast, Çintesun et al. (2021) in a study that were paid to examine the effect of vaginal progesterone suppository administration on uterine artery PI, stated that there was observed no significant difference between the groups in the right and left uterine arteries PI values, and as a result, progesterone does not have a significant effect on uterine artery PI [21].

The reason for this contradiction may be related to the smaller statistical volume of the studied samples and the short follow-up time of women in the study by Çintesun et al. Also, regarding the RI values in our study, it was clear that in the intervention group compared to the control group, the RI values after the study were significantly lower. Also, in the intervention group, the RI values after the study had greater decreased before the study compared to the control group. In a similar study, Bachar et al. (2023) stated that a single dose of 200 or 400 mg of vaginal progesterone in women with a gestational age of 24-33 weeks with diagnosed preterm birth, significantly reduced uterine, umbilical, and fetal vascular resistance after 48 hours [22].

These results were consistent with the results of our study. However, in a study which was conducted by Adan et al. (2024), by the examination of the relationship between first-trimester serum progesterone levels and uterine artery Doppler findings with adverse perinatal outcomes, it was showed that there was no statistically significant difference between serum progesterone levels with RI and S/D in 86 pregnant women without complications and 27 pregnant women with complications [17]. These findings were not consistent with the results of our study.

The reason for this discrepancy may be related to the fact that in our study, progesterone treatment was performed; however, in the study by Adan et al. (2024), only a descriptive relationship between serum progesterone levels and uterine artery Doppler findings has been examined. Whereas in our study, the use of vaginal progesterone was associated with a statistically significant reduction in uterine artery PI and RI values, particularly after the intervention, with robust effect sizes and high statistical power. These findings suggest improved uteroplacental perfusion, which may underlie the observed reduction in preterm birth.

Prior research has indicated that elevated uterine artery Doppler indices, particularly PI and RI, are associated with adverse outcomes such as preeclampsia, fetal growth restriction (FGR), and stillbirth. Studies have proposed threshold values—such as a PI>1.45 or RI >0.58 in the second trimester—as predictors of these complications [23][24]. In our trial, the post-intervention PI and RI values in the progesterone group fell below these thresholds, suggesting a potential protective effect. However, while our study did not show statistically significant reductions in other clinical outcomes beyond preterm birth (e.g., preeclampsia or FGR), this may be due to the limited sample size and event rates. Future studies with larger cohorts are needed to confirm whether reductions in uterine artery indices translate to consistent improvements in a broader range of maternal and neonatal outcomes. Notably, some previous studies using progesterone did not observe significant vascular changes [25], which may be due to differences in timing, dosing, or patient selection. Our findings support the hypothesis that vaginal progesterone may exert vascular-modulating effects in high-risk pregnancies.

While our findings suggest a beneficial effect of vaginal progesterone on uterine artery Doppler indices and preterm birth reduction, it is important to acknowledge that some previous studies have reported no significant improvements in uterine perfusion or preeclampsia prevention with progesterone use [26][27]. These discrepancies may be attributed to differences in progesterone formulations, dosages, routes of administration, timing of intervention, or variations in the studied populations’ risk profiles. For instance, studies that used oral or injectable progesterone, initiated treatment later in pregnancy, or included women at lower risk may have failed to demonstrate benefit. Moreover, our findings align with several systematic reviews indicating that vaginal progesterone may reduce the risk of preterm birth in selected high-risk populations but that its effect on preeclampsia remains less conclusive [28]. Further large-scale, well-designed trials are needed to clarify these inconsistencies.

In this study, as like as other studies, there are many known and unknown factors (such as genetics, activity, and diet type) that are limitations of this study and can affect the results of this study, and certainly, the investigation all of these cases requires more time and accuracy. Also, according to the small statistical samples size, the breadth of the subject, and the limitations of the measurement tool, any definitive opinion on the results of this study requires further and more extensive studies, which are recommended for other researchers to conduct it. According to the post-hoc power analysis was performed for the comparison of preeclampsia history between the control and intervention groups, low statistical power was obtained. This low power suggests a substantial risk of Type II error, which we have now acknowledged it as a limitation of this study. Therefore, we recommend future studies with larger sample sizes to confirm these findings. One limitation of the present study is the lack of long-term follow-up on maternal cardiovascular outcomes and neonatal development. Although we observed short-term improvements such as reduced preterm birth and favorable changes in uterine artery Doppler indices, it remains unclear whether these translate into sustained health benefits postpartum. Future longitudinal studies are warranted to evaluate the long-term clinical impact of progesterone therapy on maternal vascular health and child developmental outcomes.

## Conclusion

The results of the present study showed that treatment with 80 mg of enteric-coated oral aspirin tablets along with 400 mg of vaginal progesterone suppositories daily from the 12th week of pregnancy for 6 weeks was reduced the prevalence of preterm birth and reduced the uterine artery PI and RI values . Therefore, simultaneous administration of these two drugs in 11 to 14 weeks pregnant women with one of the high risk factors for preeclampsia could be effective in improving uterine artery vascular indices and, consequently, facilitating effective decision-making in clinical management.

## Conflict of Interest

The authors had no conflict of interest in conducting the present study
